# Oral Health Status in Older People with Dementia: A Case-Control Study

**DOI:** 10.3390/jcm10030477

**Published:** 2021-01-27

**Authors:** Pia Lopez-Jornet, Carmen Zamora Lavella, Eduardo Pons-Fuster Lopez, Asta Tvarijonaviciute

**Affiliations:** 1. Department Stomatology School of Medicine, Biomedical Research Institute (IMIB-Arrixaca), Faculty of Medicine and Odontology, University of Murcia, Adv Marques de los Velez s/n, 30008 Murcia, Spain; 2. Faculty of Medicine and Odontology, University of Murcia, 30008 Murcia, Spain; czamoral@hotmail.es (C.Z.L.); eduardo.p.f@um.es (E.P.-F.L.); 3. Interdisciplinary Laboratory of Clinical Analysis, Interlab-UMU, Regional Campus of International Excellence ‘Campus Mare Nostrum’, University of Murcia, Espinardo, 30100 Murcia, Spain; asta@um.es

**Keywords:** tooth loss, dementia, age, oral hygiene, oral health

## Abstract

Dementia is characterized by a range of cognitive defects with impaired activities of daily living that have implications for patient oral health. Objectives. A case-control study was made of the impact of dementia upon oral health. A total of 152 patients were included: 69 with dementia and 83 controls from the region of Murcia (Spain). The Global Deterioration Scale (GDS) was used to classify the patients and an oral exploration was carried out. Odds ratios (ORs) and confidence intervals (CIs) were estimated using regression models. The patients with more severe disease were significantly more likely to have fewer natural teeth (OR 11.00, 95%CI 1.28–23.22; *p* = 0.001), a higher plaque index (*p* = 0.001), and a greater bleeding index (*p* = 0.001) than the control group. These findings suggest that older adults with dementia have deficient oral health. A higher bleeding index increases the risk of deterioration of cognitive function. The oral hygiene and health of older people with dementia need to be improved.

## 1. Introduction

Dementia is characterized by a range of cognitive defects with memory loss that result in different health problems. The disorder encompasses conditions such as Alzheimer’s disease (AD), vascular dementia (VD), and other less frequent forms of dementia such as Lewy body dementia, frontotemporal lobe degeneration, or dementia associated with Parkinson’s disease [1,2,3,4,5].

There is increasing consensus that the production and accumulation of amyloid-β peptide are central to the pathogenesis of Alzheimer’s disease. Many years before the onset of Alzheimer’s symptoms, two neuropathological events occur in the brain that currently confirms the diagnosis of the disease: The formation of neurofibrillary tangles of the tau protein and the accumulation of plaques of the amyloid-β peptide [1,2].

Dementia has a growing presence with advancing age, and the associated cognitive defects with impaired activities of daily living have implications for patient oral health. Different studies have confirmed this association between cognitive impairment and poor oral health, difficulties for the correct cleaning of dentures, and dental caries [6,7,8,9,10,11,12,13]. One possible way that links both diseases (dementia and oral diseases) could be the invasion of the brain tissue by periodontal bacteria that reside in the dental biofilm (i.e., *Aggregatibacter actinomycetescomitans*; *Tannerella forsythia*, *Porphyromonas gingivalis,* and *Treponema denticola*) and their products [4]. This invasion may occur through the bloodstream or via peripheral nerves and would accelerate neuroinflammation. Periodontal-derived cytokines could also reach the brain by either systemic or neural via, thus increasing brain cytokine levels [4,9].

Tooth loss has a strong negative impact upon the oral health-related quality of life of older adults. Such tooth loss may be the result of new or recurrent caries, periodontal disease, traumatisms, or neglected oral hygiene. A growing number of studies have focused on the relationship between oral health and cognitive status [14,15,16,17,18,19,20,21,22,23].

In particular, research has centered on the association between the number of teeth in the mouth and cognitive function, though the results of the longitudinal observational studies on the effect of tooth loss upon cognitive function have been inconsistent. A systematic review carried out by Cerutti-Kopplin [24] suggested that tooth loss is associated with an increased risk of cognitive impairment and dementia, while Wu et al. [25] did not find tooth loss to be consistently associated with such disorders. These discordant findings may be attributable to methodological issues such as the representativeness of the study population, the way in which cognitive function is assessed, or the oral explorations made.

The existing evidence of a causal relationship between tooth loss and the development of dementia is limited. We, therefore, decided to carry out the present case-control study to assess the impact of dementia upon oral health.

## 2. Material and Methods

A cross-sectional descriptive study representative of the region of Murcia (Spain) was carried out, involving males and females aged 65 years or older. Simple random sampling was used to recruit 69 patients diagnosed with dementia and 83 controls from a number of homes for the elderly—Los Almendros (Cartagena), Casa de Campo (Cartagena), and Caser (Lorca)—and the Alzheimer associations AFADE Alcantarilla (Murcia), AFAL Cartagena and district (Cartagena), and AFA (Lorca).

The diagnosis of dementia was obtained from the facilities in charge of the patients (homes for the elderly or Alzheimer associations). The Global Deterioration Scale (GDS) of Reisberg was used to stage the patients [26] as follows:

1. GDS 1: Normal individual
2. GDS 2: Very mild cognitive impairment/benign senile memory loss
3. GDS 3: Mild cognitive impairment/incipient dementia disease
4. GDS 4: Moderate cognitive impairment/mild dementia
5. GDS 5: Moderately severe cognitive impairment/moderate dementia
6. GDS 6: Severe cognitive impairment/moderately severe dementia
7. GDS 7: Very severe cognitive impairment/severe dementia

All the participants and/or their relatives or legal representatives signed the corresponding informed consent document before the study was started. The study was carried out in abidance with the recommendations of the Declaration of Helsinki and was approved by the Ethics Committee of the University of Murcia (ID: 2287/2019 Murcia, Spain). The study followed the STROBE (Strengthening the Reporting of Observational Studies in Epidemiology) guidelines.

Inclusion criteria: Patients aged 65 years or older, with a confirmed diagnosis of dementia; residents in the region of Murcia; patients agreeing to and able to participate in the study.

Exclusion criteria: Patients receiving or who had received chemotherapy or radiotherapy of the head and neck; serious personality disorders; schizophrenia; drug abuse.

The controls, in turn, consisted of patients of the same sociocultural level as the patients and with the same age and gender distribution, without dementia or cognitive impairment, and with no neurodegenerative disease. All were functionally and socially active, with a Mini-Mental State Examination (MMSE) score of ≥28.

All oral explorations were made by the same trained and calibrated dentist (CZL). Demographic data were recorded, along with smoking habits, alcohol consumption, frequency of tooth brushing, and type of brush used (manual or electric). The clinical examination included an evaluation based on the protocol of the World Health Organization (1997) [27]. Then, based on the number of remaining teeth, participants were classified into 3 groups (1–9, 10–19, and 20–28 teeth) [28]. The oral hygiene was evaluated to the O’Leary plaque. The percentage of plaque-positive surfaces was calculated [29]. We recorded bleeding (bleeding index) upon probing and periodontal pocket depth (measured from the gingival margin to the base of the periodontal pocket) using a Cp11 millimetered periodontal probe; (Hu-Friedy, Germany). The organoleptic assessment of halitosis was performed using the 10-point organoleptic scale 0: No detectable odor 10: Extremely strong odor. Mouth odor (smelled at 10 cm from the oral cavity: While the patient normally breaths and while the patient counts loudly to 10) [30].

The SPSS version 20 statistical package (SPSS Inc., Chicago, IL, USA) was used throughout. All variables were tested with the Kolmogorov–Smirnov test to determine whether their distributions were normal. A descriptive study was made for each variable as mean (standard deviation, SD) or median (interquartile range, IQR). Comparison of the two groups was made applying the Student’s *t*-test or chi-square test as appropriate. The multilevel univariate logistic regression model was used to establish the effect of the demographic and clinical variables with the number of teeth in the patients, having a reference range between 20–32 pieces. The binary logistic regression was used to determine the effect of the related factors of conditions in the buccal cavity with dementia prognosis. Statistical significance was established as *p* < 0.05.

## 3. Results

The final study sample consisted of 152 subjects (98 females [64.5%] and 54 males [35.5%]), with a mean age of 75.6 ± 7.2 years (range 65–93). There were 69 patients (45.4%) and 83 controls (54.5%). The characteristics of each group are shown in Table 1 and Table 2.

The descriptive and comparative analysis of the oral health variables is shown in Table 3. The percentage of individuals who were dependent on tooth brushing was greater in the patient group than in the control group. With regard to the type of brush used, manual brushes were used by all the patients, compared with 69.9% of the controls. On the other hand, the percentage of individuals that brushed their teeth three or four times a day was significantly higher among the patients than in the control group, while the percentage of patients wearing dentures was greater than in the control group (47.8% versus 21.7%, respectively). With regard to plaque index, bleeding upon probing and pocket depth, the mean scores were significantly higher among the patients than in the control group. The patients with dementia had fewer teeth in the mouth than the controls.

With regard to the number of teeth present in the mouth, Table 4 shows the results of the parametric bivariate statistics and logistic regression models exploring the influence of the demographic and clinical variables upon the number of teeth, establishing as reference interval the presence of 20–32 teeth. The patients with severe dementia were more likely than the controls to have fewer than 20 teeth in the mouth. Specifically, they were 11 times more likely to have 1–9 teeth and 11.59 times more likely to have 10–19 teeth than the controls. The degree of dependency for tooth brushing also yielded statistically significant results. Specifically, the totally dependent patients were 33 times more likely to have 1–9 teeth and 13.38 times more likely to have 10–19 teeth than the patients that did not require help for tooth brushing. Lastly, significant results were obtained in relation to plaque index and bleeding on probing, with higher plaque and bleeding scores being associated with a greater probability of having fewer than 20 teeth in the mouth.

Data from Table 5 show the results of the multivariant regression model used to determine the effect of the related factors of conditions in the buccal cavity with dementia prognosis. Results displayed that the “times of brushing” had a significant effect, rising by 5.5 times the probability of dementia in those who never brushed their teeth (OR = 5.5) and by 3 times on those who brushed their teeth between once or twice (OR = 3). Results showed that higher bleeding increased the risk of dementia (OR = 1.16).

## 4. Discussion

Cognitive impairment and dementia are important problems in aging populations. In the present study, we found the oral health of people with dementia to be poor, and the degree of dementia was seen to play an important role in this respect—severe dementia being associated with a greater probability of having fewer teeth in the mouth than among the controls. These findings are consistent with those of other studies [31], though the concrete type of dementia involved (e.g., associated with Alzheimer’s disease, vascular dementia) does not appear to be a determinant factor in relation to oral health [32]. The severity of cognitive impairment does play a role in the oral health of older people with dementia, with increasing cognitive impairment being associated with greater plaque scores and oral disease [17,19,20]. Stein et al. [21] explored the relationship between dementia risk and the number of teeth present in the mouth and found that tooth loss may constitute an early marker of cognitive and physical deterioration.

On the other hand, it has been suggested that oral health may be related to cognitive status through the possible mediation of a common inflammatory pathway [33,34,35]. There are a number of potential mechanisms whereby tooth loss may have a negative effect on cognitive function. Periodontitis is one of the main causes of tooth loss and is able to increase the plasma concentrations of proinflammatory mediators such as IL-1, IL-6, and TNF-α, thereby contributing to worsening neuroinflammatory processes in the brain and ultimately resulting in cognitive impairment. Furthermore, chewing problems caused by tooth loss can result in deficient nutritional conditions with decreased cerebrovascular flow—the latter possibly being related to memory defects [35,36,37,38,39]. Kato et al. [22] recently found that the maintenance of dental health and/or the use of artificial teeth may help preserve cognitive function in older adults.

Thomson and Barack [40] suggested that the possible related mechanisms between tooth loss and cognitive decline in older people are: (1) Tooth loss can compromise nutritional status and this can lead to a weakened nervous system. (2) Tooth loss results in less “interoclusal contacts”, therefore, less somatosensory feedback, leading to a cognitive decline, and (3) chronic periodontitis drives tooth loss, and during this inflammatory process, CNS can be affected, as so can cognition.

Tooth loss reflects the history of diseases, health-related habits, and attention to health in the course of the lifetime of an individual. Good oral health, therefore, requires daily plaque elimination by brushing and the care of oral hygiene among dependent older individuals [15,17,21,23]. The degree of dependency for tooth brushing was also seen to have a statistically significant influence. Specifically, totally dependent people were seen to be 33 times more likely to have 1–9 teeth in the mouth than the controls—though it must be emphasized that oral health can be maintained through good assisted oral hygiene.

Patients with dementia suffer an increased presence of oral disease [23]. On the one hand, the loss of individual skills among such individuals results in defective hygiene, while on the other, the drug treatments they typically receive result in salivary dysfunction, with an increased incidence of caries [13,14,15,16,17,18,19,20]. The plaque index and bleeding upon probing scores likewise yielded statistically significant results, with higher scores increasing the probability of having fewer than 20 teeth in the mouth.

It is important for medical reasons to minimize the sources of microorganisms in the mouth, as these may, in turn, affect other parts of the body. In this regard, dental plaque is the main cause of gingivitis, and subsequently of periodontitis [7,8,9,10,11,12,35,39]. Therefore, an important plaque index presence in older people with dementia can be expected to result in high incidences of gingivitis and periodontitis. In this regard, periodontitis has been associated with multiple systemic disorders, and the evidence found in the literature supporting the etiopathogenic role of these microorganisms is too strong to be ignored as a mere coincidence [35,36,37,38,39].

The contradictory findings referring to the relationship between dementia and tooth loss can be explained by methodological problems referred to a lack of calibration of the dental evaluations and the absence of standard oral health examination protocols such as those used in our study. On the other hand, one of the limitations of the present study is its cross-sectional design, and also the patients and controls could suffer other different diseases that could interfere with oral health. Further interventional studies involving long-term follow-up periods are, therefore, indicated.

In the general population, the widespread lack of awareness of the importance of oral health explains in part the prevalence of tooth loss [37]. Given the importance of oral health in relation to the risk of cognitive impairment, oral health education programs are needed. It is important to underscore that physicians must be aware of this association and that oral exploration should form part of the integral evaluation of individuals with a high risk of suffering dementia [10,11,12,13,14].

The oral health and hygiene of older people with dementia need to be improved [40,41,42]. This could be achieved through caregiver education in oral health, the use of oral disease detection tools, the assistance of dental professionals in adequate oral care and correct treatment planning, behavioral management of dementia patients, and the intervention of multidisciplinary health professional teams [4,5,6,7,8].

Lastly, poor oral health may possibly constitute a marker of individual health in general, in addition to acting as a possible risk factor for dementia among older people. In this respect, the association between oral health and dementia risk is an important global concern for maintaining an adequate quality of life.

## 5. Conclusions

These findings suggest that older adults with dementia have deficient oral health. The higher bleeding index increases the risk of deterioration of cognitive function. The oral hygiene and health of older people with dementia need to be improved

## Figures and Tables

**Table 1:** Description and comparison of demographic variables and habits of the two study groups.

	Group		*p*-Value
	Controls	Patients	
Age	74.82 (8.17)	76.54 (5.65)	0.142
Gender			0.868
Female	54 (65.1)	44 (63.8)	
Male	29 (34.9)	25 (36.2)	
Educational level			0.02
none	16 (19.3) a	17 (24.6) a	
Primary	28 (33.7) a	22 (31.9) a	
Secondary	22 (26.5) a	27 (39.1) a	
University	17 (20.5) a	3 (4.3) b	
Smoking			0.064
Yes	20 (24.1)	8 (11.6)	
No	43 (51.8)	35 (50.7)	
Ex-smoker	20 (24.1)	26 (37.7)	
Alcohol			0.022
No	51 (61.4) a	45 (65.2) a	
Once a week	7 (8.4) a	5 (7.2) a	
Weekend	13 (15.7) a	18 (26.1) a	
Daily	12 (14.5) a	1 (1.4) b	

a,b Significant difference compared with patients group/control group. Statistical significance was established as *p* < 0.05.

**Table 2:** Description and comparison of clinical variables of the two study groups.

	Group		*p*-Value
	Controls	Patients	
No. drugs	4.81 (3.45)	5.39 (2.18)	0.225
BMI	26.95 (3.77)	28 (4.4)	0.281
Cardiovascular disease	48 (57.8)	39 (56.5)	0.871
Endocrine disease	48 (57.8)	35 (50.7)	0.381
Respiratory disease	17 (20.5)	26 (37.7)	0.019
Locomotor disease	47 (56.6)	48 (69.6)	0.101
Gastrointestinal disease	30 (36.1)	15 (21.7)	0.049
Hematological disease	29 (34.9)	30 (43.5)	0.282
Urogenital disease	30 (36.1)	42 (60.9)	0.002

BMI: Body mass index. Statistical significance was established as *p* < 0.05.

**Table 3:** Description and comparison of oral health variables of the two study groups.

	Group		*p*-Value
	Controls	Patients	
Halitosis score, n (%)			0.202
1	28 (33.7)	15 (21.7)	
2	18 (21.7)	20 (29)	
3	26 (31.3)	18 (26.1)	
4	8 (9.6)	12 (17.4)	
5	3 (3.6)	2 (2.9)	
6		2 (2.9)	
Independence for brushing, n (%)			<0.001
Alone	72 (86.7)	45 (65.2)	
Help	11 (13.3)	14 (20.3)	
Dependent		10 (14.5)	
Type of brush, n (%)			<0.001
Manual	58 (69.9)	69 (100)	
Electric	25 (30.1)		
No. brushings/day, n (%)			0.021
1	29a (34.9)	17a (24.6)	
2	24a (28.9)	23a (33.3)	
3	14a (16.9)	3b (4.3)	
4	14a (16.9)	22b (31.9)	
Never	2a (2.4)	4a (5.8)	
Dentures, n (%)			0.001
No	65 (78.3)	36 (52.2)	
Yes	18 (21.7)	33 (47.8)	
Denture condition, n (%)			0.273
Good	12 (70.6)	18 (54.5)	
Poor	5 (29.4)	15 (45.5)	
Pocket depth, mean (SD)	2.57 (0.98)	3.05 (0.99)	0.004
Index plaque, mean (SD)	44.85 (16.84)	57.75 (12.66)	<0.001
Bleeding upon probing, mean (SD)	54.72 (19.57)	70.97 (14.48)	<0.001
No. teeth in mouth, mean (SD)	19.23 (6.91)	14.2 (7.93)	<0.001

Statistical significance was established as *p* < 0.05.

**Table 4:** Influence of the demographic and clinical variables upon the number of teeth.

	Number of Teeth in Mouth			
	1–9		10–19	
	OR (95%CI)	*p*-Value	OR (95%CI)	*p*-Value
GSD				
Control	Ref.			
Mild	8.62 (1.95–38.03)	0.004	1.53 (0.62–3.80)	0.361
Moderate	2.29 (0.21–25.15)	0.499	2.13 (0.72–6.33)	0.174
Severe	11.00 (1.28–23.22)	<0.001	11.59 (1.36–29.01)	0.025
Gender				
Male	Ref.			
Female	1.87 (0.55–6.30)	0.315	0.79 (0.38–1.63)	0.52
Educational level				
University	Ref.			
None	6.92 (0.73–65.26)	0.091	2.60 (0.65–10.45)	0.179
Primary	5.63 (0.60–52.37)	0.129	5.83 (0.79–22.41)	0.064
Secondary	3.00 (0.32–28.19)	0.337	2.55 (0.72–9.02)	0.146
Smoking				
No	Ref.			
Yes	2.55 (0.73–8.93)	0.142	1.62 (0.72–3.67)	0.247
Ex-smoker	0.29 (0.03–2.82)	0.286	0.58 (0.20–1.65)	0.306
Alcohol consumption				
No				
Occasional	1.04 (0.91–2.04)	0.887	1.06 (0.84–2.55)	0.954
Daily	1.94 (1.21–4.04)	0.121	1.02 (0.87–1.91)	0.916
Independence for brushing				
Alone	Ref.			
Help	17.71 (3.69–25.96)	0.002	8.92 (1.23–19.25)	0.043
Dependent	33.00 (5.71–45.66)	<0.001	13.38 (2.90–21.74)	0.001
No. brushings/day				
Never	Ref.			
1–2	0.12 (0.01–2.09)	0.144	0.20 (0.02–2.00)	0.17
3–4	0.69 (0.04–12.20)	0.798	0.48 (0.05–5.03)	0.54
Plaque index	1.39 (1.07–1.47)	<0.001	1.18 (1.03–1.10)	<0.001
Bleeding on probing	1.26 (1.06–1.15)	<0.001	1.15 (1.03–1.09)	<0.001

OR: Odds ratio; CI: Confidence interval; Statistical significance was established as *p* < 0.05.

**Table 5:** Effect of the factors related to oral cavity conditions in the prognosis of dementia.

	B (ET)	Wald	OR (CI 95%)	*p*-Value
No. brushings/day				
3–4				
Never	1.70 (0.61)	7.78	5.50 (1.66–18.20)	0.005
1–2	1.10 (0.51)	4.65	3.00 (1.11–8.13)	0.031
Independence for brushing				
Alone				
Help	1.89 (1.53)	1.53	6.62 (0.33–131.83)	0.216
Dependent	−0.37 (0.57)	0.41	0.69 (0.23–2.11)	0.520
Plaque index	−0.05 (0.05)	0.94	0.96 (0.87–1.05)	0.332
Bleeding on probing	0.15 (0.03)	19.84	1.16 (1.09–1.24)	<0.001
No. teeth in mouth	−0.06 (0.04)	2.59	0.94 (0.88–1.01)	0.108

Model: χ^2^(7) = 46.18, *p* < 0.001. R^2^ Nagelkerke = 36.7. Statistical significance was established as *p* < 0.05.

## Data Availability

This research has been approved by the Ethics Committee of the University of Murcia (approval ID: 2287/2019).
